# Ambient Scribe Technology in Simulated Patient Encounters Across Specialties

**DOI:** 10.1001/jamanetworkopen.2025.52870

**Published:** 2026-01-07

**Authors:** Julian Brunner, Suzanne Morrissey, Elise M. Stevens, Chína Payne, Scott Wiltz, Sarah L. Cutrona, Seppo T. Rinne

**Affiliations:** 1 Center for the Study of Healthcare Innovation, Implementation & Policy, Veterans Affairs (VA) Greater Los Angeles Healthcare System, Los Angeles, California; 2 Center for Health Optimization & Implementation Research, VA Bedford Healthcare System, Bedford, Massachusetts; 3 Department of Population and Quantitative Health Sciences, UMass Chan Medical School, Worcester, Massachusetts; 4 Simulation Learning, Evaluation, Assessment, and Research Network, Veterans Health Administration, Orlando, Florida; 5 Pulmonary and Critical Care Medicine, Dartmouth-Hitchcock Medical Center, Lebanon, New Hampshire

## Abstract

This qualitative study evaluates ambient scribe technology using standardized patient encounters in a simulated clinical environment, combining a validated measure of documentation quality with qualitative insights.

## Introduction

Ambient scribe technology, which generates proposed clinical documentation from patient-clinician conversations, has emerged as a promising way to reduce documentation burden. While studies on ambient scribing proliferate,^1^ rigorous independent evaluations in controlled settings are scarce, and comprehensive assessments comparing performance across medical specialties are largely absent. To address these evidence gaps, we conducted a simulation-based evaluation of ambient scribing across 4 medical specialties. We evaluated the technology using standardized patient encounters in a simulated clinical environment, combining a validated measure of documentation quality with qualitative insights.

## Methods

We conducted a mixed-methods study from February 4 to 6, 2025, at the Veterans Health Administration’s simulation center, simultaneously testing large language model–based ambient scribing solutions (A and B). Sixteen specialist clinicians (4 each from cardiology, gastroenterology, hematology-oncology, and neurology) participated in multiple simulated patient encounters with standardized patients enacting specialty-specific clinical scenarios (eTable 1 in Supplement 1), with each specialty including scenarios with intentional challenges (eg, involvement of a translator or family member, noisy intrusions from third parties). Each encounter was recorded simultaneously by both ambient scribing solutions, which then generated clinical notes. Specialists completed an adapted Physician Documentation Quality Instrument (PDQI-9)^2^ (eTable 2 in Supplement 1) for each note and participated in specialty-specific focus groups (eTable 3 in Supplement 1). We calculated means and SDs for PDQI-9 scores and used analysis of variance to assess differences among specialties. This project was reviewed by the Veterans Affairs Bedford Institutional Review Board, which designated it as nonresearch not subject to informed consent requirements. We conducted thematic analysis of focus group transcripts to contextualize quantitative findings, with qualitative methods adhering to the COREQ guideline.

## Results

The mean (SD) documentation quality rating was 36.2 (10.9) of 50; ratings were highest for succinctness and lowest for thoroughness (Table 1). Across specialties, summary PDQI-9 scores were highest for neurology (42.2 [7.5]) and lowest for cardiology (32.0 [10.0]), with gastroenterology (36.8 [12.7]) and hematology-oncology (35.0 [11.3]) between; differences did not reach statistical significance (ANOVA, *P* = .09).

**Table 1.** Mean Ambient Scribing Ratings

PDQI-9 item	Mean (SD) score^a^					
	Neurology	Cardiology	Gastroenterology	Hematology/oncology	Total
Accurate	4.4 (1.2)	3.3 (1.2)	3.5 (1.2)	3.4 (1.1)	3.6 (1.2)
Thorough	3.8 (1.2)	2.8 (1.2)	3.5 (1.3)	3.5 (1.2)	3.4 (1.2)
Useful	3.9 (1.1)	3.3 (1.1)	3.8 (1.4)	3.5 (1.2)	3.6 (1.2)
Organized	4.2 (1.3)	2.7 (1.3)	3.7 (1.5)	3.5 (1.3)	3.5 (1.3)
Comprehensible	4.1 (1.1)	3.1 (1.1)	3.7 (1.4)	3.4 (1.4)	3.5 (1.2)
Succinct	4.3 (1.4)	3.8 (1.4)	4.0 (1.4)	3.6 (1.3)	3.9 (1.2)
Synthesized	4.0 (1.2)	3.2 (1.2)	3.9 (1.3)	3.3 (1.3)	3.6 (1.2)
Internally consistent	4.2 (1.1)	3.3 (1.1)	3.7 (1.5)	3.6 (1.2)	3.7 (1.2)
Free from hallucination	4.0 (1.3)	3.3 (1.3)	3.5 (1.6)	3.2 (1.3)	3.5 (1.4)
Free from bias	4.5 (1.3)	3.3 (1.3)	3.7 (1.5)	3.9 (1.3)	3.8 (1.3)
Summary score	41.2 (7.5)	32.0 (10.0)	36.8 (12.7)	35.0 (11.3)	36.2 (10.9)

Abbreviation: PDQI-9, Physician Documentation Quality Instrument.

^a^ Includes 82 respondents. Item scores range from 0 to 5 and summary scores range from 0 to 50, with higher scores indicating higher-quality documentation rating.

The mean ratings were 38.3 (5.8) for solution A and 33.1 (11.9) for solution B, yet qualitative feedback suggested that when solution B overcame technical reliability challenges, specialists frequently preferred the organization of its output. Further qualitative analyses provide context, highlighting perceived advantages, concerns, and specialty-specific needs that may affect the utility of ambient scribing (Table 2). For example, neurologists described discussion-heavy encounters in which ambient scribing helped maintain eye contact and capture details that might otherwise be missed, while cardiologists emphasized the centrality of prior studies and medical record data.

**Table 2.** Perceived Advantages, Concerns, Specialty Differences, and Key Use Cases For Ambient Dictation

Category by theme	Description	Sample quotation [specialty participant identification No.]
**Advantages**		
Saving time and mental load	Participants valued the ability of ambient scribing to offload the most labor-intensive part of note-writing, even if the proposed notes were typically inadequate on their own.	Since it takes so much time to generate a note anyway, this would decrease time for the simple reason that we wouldn’t have to type the note [neurology 01].
Catching details	Specialists noted that ambient scribing could improve the quality of their documentation by making it easier to capture all salient information covered in an examination, and by allowing them to review patient comments they may have otherwise overlooked.	It allows me to not forget, “oh, I did ask that question, and I don’t document it” ... It’ll help me to avoid missing out on important documentation [gastroenterology 02].
Improving eye contact	Specialists observed that ambient dictation could help clinicians keep their eyes directed at the patient instead of focused on a computer interface.	Neurologists hate looking at the typing screen, they like to look at the eye contact, what the patient does, their mannerisms, etc [neurology 01].
**Concerns**		
Need for EHR integration	Specialists emphasized that ambient scribing solutions must connect with prior notes, diagnostic results, and other patient data to be truly valuable.	I think capturing the dictation and the interaction with the patient is 1 piece, but how it’s going to integrate with the EMR and will it have the capability where you can sort of build a template, which is what we’re used to doing in our notes currently, where we tell it, you know, pull the most recent echo[cardiogram], pull the most recent cath, pull the most recent cardiac CT. So that integration piece, I think, is going to be key to seeing if this can be successful in specialty care [cardiology 01].
Inaccuracies and hallucinations	Specialists also emphasized the hazards of widespread inaccuracies and hallucinations in the proposed notes, including fabricated physical examination findings, failure to attribute information sources, inaccurate *CPT* codes, and omissions of pertinent negatives.	Some of the stuff that we saw here was just wrong. Like, it said that something happened 12 years ago when it happened in 2017, and that wasn’t 12 years ago. Or [for] allergies—it said “none,” and it wasn’t asked. Like, those are big safety issues [hematology/oncology 01].
Diagnostic certainty	Specialists were concerned by a tendency of the technology to offer diagnoses with more certainty and specificity than appropriate, without properly representing real ambiguity.	The other issue is the confidence that both of the programs have in the diagnosis. A lot of neurologic diseases are clinical diagnoses without a specific biomarker that gives you a definitive diagnosis. So a lot of times we’re hedging a little bit. It’s probably this, but it could be this or that. There’s really a differential. And the programs are putting up very specific “it’s this.” At the most, in one of the notes I saw, it put “suspected” next to it. But it didn’t give a whole differential “Oh, it could be MS, it could be NMO, it could be MOGAD, it could be Lyme disease. It’s just MS” [neurology 03].
Distorting the physical examination results	Specialists noted that natural patient-facing speech would typically fail to capture the information needed to document a physical examination finding and were concerned that documentation needs would degrade verbal communication with patients.	The thing that I struggle with is changing the way I act with the patient.... It builds a little bit of distance from the patient in terms of now I’m becoming more robotic than a human. So that’s the part that I can’t get past [gastroenterology 01].
Privacy concerns	Specialists expressed speculative concerns about privacy and impacts of ambient scribing on patients’ willingness to disclose sensitive information.	That might cause patients to leave out certain parts of what they may want to tell us. Like, “I was incarcerated last month ... and while I was incarcerated, the doctor had to come visit me for whatever,” but he might just eliminate that whole thing completely. And then there’s, in a doctor-patient visit, patients do say a lot of personal things. And I think that helps in sort of bond building with your physician who you see repeatedly. And I don’t know if they would be sort of on guard when they know that these things are being recorded [cardiology 01].
**Differences by specialty**		
Neurology perceived the most value	Neurologists highlighted the potential for ambient dictation to allow them to maintain eye contact with patients and capture details of patient history that they might otherwise fail to record.	I see a lot of utility for this kind of tool to be available for us to use in clinic, especially in neurology, where we like to maintain eye contact with the patient and have a lot of one-to-one discussion. And we spend a lot of time on counseling. And so with this time-based billing, having all those elements transcribed into the note and accounted for us to bill properly would be helpful [neurology 02].
Cardiology perceived the least value	Cardiologists were least enthusiastic about the technology: identifying narrow situations in which it would be helpful and emphasizing that without integration of information from the EHR, it would be unlikely to make their work easier or more efficient.	I think using just what is dictated is never going to be sufficient for me for my documentation. I feel like there’s always going to need to be some either extrapolated content from chart that is a baseline format that leads to your baseline note, because I integrate so many of the imaging tests that have previously been done, prior blood work, prior additional testing. I feel like this note to me, if I were to use this alone, if it wasn’t nicely broken down, integrated into your EMR ... would be more cumbersome and time consuming.... I mean, I went through a full substance use review, and it doesn’t document that, that I reviewed all illicit substances. Because if it’s not there, sometimes you assume you didn’t ask, right? And so the pertinent negatives were not on [cardiology 03].
Gastroenterology and hematology/oncology perspectives were mixed	Gastroenterologists and hematology-oncology specialists highlighted the value of reduced typing while interacting with patients but expressed concerns about inaccuracies and oversimplifications.	Would I rather wait a year or two for something better rather than being stuck for 10 years with something bad? I mean, I’d rather wait a year or two [for a better solution]. But ... I’d rather have this than nothing. Because you could always not use it [hematology-oncology 01].
**Key use cases**		
Good for subjective	Specialists suggested that the tools seemed most useful for generating the subjective portion of the visit, and especially the history of present illness, also highlighting new patient assessments as more suitable than follow-up visits.	The place where this would be useful would be for capturing my—just the subjective portion of the history. The rest of this, it’s like, I’m not sure that this would change my workflows a lot, precisely because there is no way to use this to capture the information that’s previously in there. It would actually create more work for me [cardiology 04].
Desired use cases	Specialists highlighted clinical decision support, after-visit summaries, and documentation during procedures as high-potential future directions for the technology.	I don’t think any of these are billing themselves as decision support tools. They’re merely ambient dictation. But there’s a great opportunity for them to also offer decision support. Again, highlight it, you know, consider these other diagnoses in your differential and consider these treatment options [gastroenterology 03].

Abbreviations: *CPT*, *Current Procedural Terminology*; CT, computed tomography; EHR, electronic health record; EMR, electronic medical record; MOGAD, myelin oligodendrocyte glycoprotein antibody disease; MS, multiple sclerosis; NMO, neuromyelitis optica.

## Discussion

In this mixed-methods qualitative study of ambient scribing in simulated patient encounters across specialties, we found moderate documentation quality of artificial intelligence–generated notes, with potential differences by specialty. To our knowledge, these findings represent the first cross-specialty comparative analysis of this technology in a controlled environment.

While the sample size of our study precludes conclusions about the suitability of ambient scribing for different specialties, descriptive differences by specialty in mean ratings and qualitative assessments of the tools are informative. In particular, the differing descriptions provided by neurologists and cardiologists highlight the need for integration of electronic health records.

Our mean documentation quality score of 36.2 compares unfavorably with the performance reported for clinical ambient scribe deployment.^2,3^ However, ratings in our study were similar to those seen in other first-exposure or simulation settings.^4,5^ Our findings must be understood in the context of the simulation’s goals: to apply a stress test to the technology with challenging scenarios before clinical deployment and to understand the factors that might constrain or improve it across multiple specialties.

Other limitations of the study include generalizability constraints of a controlled simulation environment, the limited subset of specialties included, and the lack of electronic health record integration in the tools assessed. Future evaluation and implementation should examine clinical performance over time, with attention to specialty-specific workflows and integration with the EHR.

## Notes

Supplement 1. **eMethods.** Procedures, Participants, and Analysis **eTable 1.** Overview of Simulated Patient Encounters **eTable 2.** Survey Instrument: Adapted PDQI-9 **eTable 3.** Focus Group Guide

Supplement 2.
Data Sharing Statement
