# Supplementary material for: Ambient Scribe Technology in Simulated Patient Encounters Across Specialties
Source: JAMA Netw Open. 2026 Jan 7;9(1):e2552870. doi: 10.1001/jamanetworkopen.2025.52870 (PMC12780926; doi:10.1001/jamanetworkopen.2025.52870)
Supplement: Supplement 1. — eMethods. Procedures, Participants, and Analysis eTable 1. Overview of Simulated Patient Encounters eTable 2. Survey Instrument: Adapted PDQI-9 eTable 3. Focus Group Guide [file jamanetwopen-e2552870-s001.pdf]

Supplementary Online Content

Brunner J, Morrissey S, Stevens EM, et al. Ambient scribe technology in simulated patient encounters across specialties. *JAMA Netw Open.* 2025;9(1):e2552870. doi:10.1001/jamanetworkopen.2025.52870

eMethods. Procedures, Participants, and Analysis
eTable 1. Overview of Simulated Patient Encounters
eTable 2. Survey Instrument: Adapted PDQI-9
eTable 3. Focus Group Guide

This supplementary material has been provided by the authors to give readers additional information about their work.

© 2026 Brunner J et al. *JAMA Network Open*.

eMethods. Procedures, Participants, and Analysis

We conducted a simulation in February 2025 at the Veterans Health Administration's Simulation, Validation, Evaluation, and Testing (SimVET) Center to assess the performance of ambient scribing technology across multiple medical specialties. We used a convergent parallel mixed-methods design incorporating surveys and focus groups to evaluate user perceptions of the technology. The study was conducted as part of an operations project designated by the Veterans' Affairs (VA) Bedford Institutional Review Board as a non-research operations activity designed to meet information needs of VA decisionmakers.

Participants

Specialists were nominated by the VA Specialty Care Program Office seeking representation from clinicians with active practices in cardiology, gastroenterology, hematology/oncology, and neurology. Participants were recruited without reference to demographic characteristics. Standardized patient scenarios representing typical outpatient presentations were developed by subject matter experts. Trained actors portrayed common clinical presentations in each specialty.

Simulation and Data Collection

Two ambient scribing solutions were used simultaneously during each patient encounter. These solutions operated in the background, recording the specialist-patient interactions and automatically creating proposed clinical notes. In several instances, technical issues resulted in failed note generation, with completion rates varying across encounters. Thirteen survey responses that documented a note generation failure (i.e., when a given solution failed to generate a note for a given simulated encounter) were excluded from analysis. This reduced the number of possible surveys from 128 (4 specialties * 4 specialists in each specialty * 4 scenarios per specialist * 2 solutions per scenario) to 115 possible surveys.

© 2026 Brunner J et al. *JAMA Network Open*.

Simulated encounters were designed to last approximately 20 minutes but were allotted up to an hour each to allow time for participants to review generated notes and complete surveys. Following each encounter, specialists completed surveys evaluating the quality of documentation produced by the ambient scribing technology using the PDQI-9 (Physician Documentation Quality Instrument-9), an adapted tool for assessing clinical documentation quality specifically for AI scribes. The instrument provides a summary score ranging from 0-50, with higher scores indicating better documentation quality across dimensions including accuracy, thoroughness, organization, and usefulness.

We conducted four 1-hour specialty-specific focus groups with four members each. All specialists had directly participated in at least two simulated encounters and observed several more prior to the focus groups. These sessions explored participants' perceptions of the technologies' strengths and limitations, specialty-specific considerations, and potential use cases. Focus group discussions were professionally transcribed for analysis.

Analysis

A total of 99 surveys were returned, 13 of which were excluded because they referred to a case whose free text comment indicated that a note failed to generate, and 4 of which were excluded due to item-level missing data, resulting in an analytic sample of n=82. This sample included 56 notes from solution A and 26 from solution B.

For qualitative analysis, focus group transcripts were analyzed using a codebook developed based on the study goals and detailed notes taken by the evaluation team. We conducted thematic analysis to identify key themes related to perceived advantages of the technology, concerns about implementation, differences across specialties, and potential use cases. Quantitative and qualitative results are presented separately, with qualitative data offering contextual insight into quantitative patterns.

© 2026 Brunner J et al. *JAMA Network Open*.

eTable 1. Overview of Simulated Patient Encounters

Specialty	Clinical Scenarios
Neurology	Migraines; Epilepsy; Multiple Sclerosis; Traumatic Brain Injury
Cardiology	Chest Pain (Outpatient); Heart Failure; Atrial Fibrillation; Aortic Stenosis
Gastroenterology	Hepatology; General GI; Ulcerative Colitis; Pre-operative procedure
Hematology/Oncology	Initial consultation; Routine follow-up; Progression of care; New blood clot

© 2026 Brunner J et al. *JAMA Network Open*.

eTable 2. Survey Instrument: Adapted PDQI-9

SIMULATION NAME/NUMBER (e.g., CardioSim1):

AMBIENT SOLUTION (Vendor 1 or Vendor 2):

Instructions: Please rate the AI-generated note on each of the following attributes:

Attribute	Score	Description of Ideal Note
**1. Accurate**	Not at all 1 2 3 4 5 Extremely	The note is true. It is free of incorrect information.
**2. Thorough**	Not at all 1 2 3 4 5 Extremely	The note is complete and documents all of the issues of importance to the patient.
**3. Useful**	Not at all 1 2 3 4 5 Extremely	The note is extremely relevant, providing valuable information and/or analysis.
**4. Organized**	Not at all 1 2 3 4 5 Extremely	The note is well-formed and structured in a way that helps the reader understand the patient's clinical course.
**5. Comprehensible**	Not at all 1 2 3 4 5 Extremely	The note is clear, without ambiguity or sections that are difficult to understand.
**6. Succinct**	Not at all 1 2 3 4 5 Extremely	The note is brief, to the point, and without redundancy.
**7. Synthesized**	Not at all 1 2 3 4 5 Extremely	The note reflects the author's understanding of the patient's status and ability to develop a plan of care.

© 2026 Brunner J et al. *JAMA Network Open*.

**8. Internally Consistent**	Not at all 1 2 3 4 Extremely 5	No part of the note ignores or contradicts any other part.
**9. Free from Hallucination***	Not at all 1 2 3 4 Extremely 5	The note is free of hallucination and only contains information verifiable by the transcript.
**10. Free from Bias***	Not at all 1 2 3 4 Extremely 5	The note is free of bias and contains only information verifiable by the transcript and not derived from characteristics of the patient or visit.
**Total Score:**		

Please use the other side of this form for any additional comments.

*"*hallucinations* (false information provided without sound basis) and *bias* (biased results based on use of discriminatory data, algorithms, or faulty heuristics)," Tierney et al. 2024.

Form adapted from: Tierney, Aaron & Gayre, Gregg & Hoberman, Brian & Mattern, Britt & Ballesca, Mamuel & Kipnis, Patricia & Liu, Vincent & Lee, Kristine. (2024). Ambient Artificial Intelligence Scribes to Alleviate the Burden of Clinical Documentation. NEJM Catalyst. 5. 10.1056/CAT.23.0404.

© 2026 Brunner J et al. *JAMA Network Open*.

eTable 3. Focus Group Guide

**Topic**	**Discussion Prompts**
Introduction	To start: tell us about using the different ambient scribe solutions in the clinical simulations so far.
Workflow	How did using these AI solutions compare to what you'd normally do when documenting patient encounters? First in terms of the process you go through, then in terms of the documentation you end up with?
Use cases	Thinking about the kind of work that providers in your specialty do the most, do these tools seem better for some clinical scenarios than for others?
General perspectives on integration	Based on your experience with these solutions so far, what are your thoughts on integrating ambient scribing in your specialty?
Conclusion	Any final thoughts you'd like to share?

© 2026 Brunner J et al. *JAMA Network Open*.
