# Supplementary material for: Ambient Scribe Technology in Simulated Patient Encounters Across Specialties
Source: JAMA Netw Open. 2026 Jan 7;9(1):e2552870. doi: 10.1001/jamanetworkopen.2025.52870 (PMC12780926; doi:10.1001/jamanetworkopen.2025.52870)
Supplement: Supplement 2. — Data Sharing Statement [file jamanetwopen-e2552870-s002.pdf]

Data Sharing Statement

Brunner. Ambient Scribe Technology in Simulated Patient Encounters Across Specialties. *JAMA Netw Open*. Published January 07, 2026. doi:10.1001/jamanetworkopen.2025.52870

Data
Data available: No

Additional Information
Explanation for why data not available: Full data from this study will not be made available to others due to the terms of agreements with the software solutions evaluated, but some additional information may be made available upon request.
